# A diagnostic model of autoimmune hepatitis in unknown liver injury based on noninvasive clinical data

**DOI:** 10.1038/s41598-023-31167-w

**Published:** 2023-03-10

**Authors:** Haiyan Yang, Lingying Huang, Ying Xie, Mei Bai, Huili Lu, Shiju Zhao, Yueqiu Gao, Jianjun Hu

**Affiliations:** 1 General Medical Department, Nanjing Drum Tower Hospital, the Affiliated Hospital of Nanjing University Medical School, Nanjing, China; 2 Department of Hepatology, Shuguang Hospital, Affiliated to Shanghai University of Traditional Chinese Medicine, Shanghai, China; 3 Department of Infectious Disease, Changzheng Hospital, Naval Medical University, Shanghai, China; 4 Department of Dermatology, Shanghai Sixth People’s Hospital Affiliated to Shanghai Jiao Tong University, Shanghai, China; 5 Engineering Research Center of Cell & Therapeutic Antibody, Ministry of Education, School of Pharmacy, Shanghai Jiao Tong University, Shanghai, China; 6 Special medical department, Shanghai Sixth People’s Hospital Affiliated to Shanghai Jiao Tong University, Shanghai, China; 7 Department of Infectious Disease, Tongren Hospital, Shanghai Jiao Tong University School of Medicine, 1111 Xianxia Road, Shanghai, 200336 China

**Keywords:** Autoimmune hepatitis, Hepatitis

## Abstract

All the diagnostic criteria of autoimmune hepatitis (AIH) include histopathology. However, some patients may delay getting this examination due to concerns about the risks of liver biopsy. Therefore, we aimed to develop a predictive model of AIH diagnostic that does not require a liver biopsy. We collected demographic, blood, and liver histological data of unknown liver injury patients. First, we conducted a retrospective cohort study in two independent adult cohorts. In the training cohort (n = 127), we used logistic regression to develop a nomogram according to the Akaike information criterion. Second, we validated the model in a separate cohort (n = 125) using the receiver operating characteristic curve, decision curve analysis, and calibration plot to externally evaluate the performance of this model. We calculated the optimal cutoff value of diagnosis using Youden’s index and presented the sensitivity, specificity, and accuracy to evaluate the model in the validation cohort compared with the 2008 International Autoimmune Hepatitis Group simplified scoring system. In the training cohort, we developed a model to predict the risk of AIH using four risk factors—The percentage of gamma globulin, fibrinogen, age, and AIH-related autoantibodies. In the validation cohort, the areas under the curve for the validation cohort were 0.796. The calibration plot suggested that the model had an acceptable accuracy (*p* > 0.05). The decision curve analysis suggested that the model had great clinical utility if the value of probability was 0.45. Based on the cutoff value, the model had a sensitivity of 68.75%, a specificity of 76.62%, and an accuracy of 73.60% in the validation cohort. While we diagnosed the validated population by using the 2008 diagnostic criteria, the sensitivity of prediction results was 77.77%, the specificity was 89.61% and the accuracy was 83.20%. Our new model can predict AIH without a liver biopsy. It is an objective, simple and reliable method that can effectively be applied in the clinic.

## Introduction

Autoimmune hepatitis (AIH) is an liver inflammation caused by abnormal autoimmunity^1^. It is still being difficult to determine because of a variety of genetic, environmental, and other factors^2,3^. In AIH early stages, symptoms are generally mild and may include mild fatigue, loss of appetite, jaundice, nausea, itching, and joint pain^4–6^. Some AIH patients have early symptoms known as “unknown liver injury (ULI)” that are not diagnosed or treated promptly and eventually progress to serious diseases like liver failure^7–9^.

Due to the heterogeneity of clinical presentation, early detection is challenging, and no single diagnostic test is suitable for all patients. The International Autoimmune Hepatitis Group (IAIHG) proposed and revised the diagnostic criteria for AIH in 1993 and 1999, respectively^10,11^. Due to its complexity, the IAIHG simplified the scoring system in 2008^12,13^. All diagnostic criteria include histopathological examination, which is crucial for the definitive diagnosis of AIH patients^14,15^. Unfortunately, in clinical practice, some ULI patients have not undergone to be exanimated due to concerns about the invasiveness and uncertainty of liver biopsy, further reducing the rate of early diagnosis and practical application value of the IAIHG simplified scoring system in 2008.

Based on this deficiency, we aimed to develop a liver biopsy-free model to predict the risk of AIH in adults and validate its accuracy.

## Methods

### Study design

This research used a cross-sectional study design. We collected the data from the electronic case system, and data were primarily collected retrospectively from December 27, 2005, to January 8, 2020, in three hospitals that belong to territory medical institutions. The study was conducted at the Shanghai Sixth People's Hospital in the Xuhui district, Shuguang Hospital in the Pudong district and Changzheng Hospital in the Huangpu district. Data from the Shanghai Sixth People’s Hospital were used to develop a model, and data from the other two hospitals were used for model validation. All patients undergoing liver biopsy were informed in advance of the relevant risks and purposes by the attending physician and signed the informed consent of this study. This study was approved by the Ethics Committees of Shanghai Sixth People’s Hospital with approval number 2020-KY-032(K). And we can confirm that all methods were performed in accordance with relevant guidelines and regulations.

### Case selection and classification

We collected demographic, clinical, biochemical, and pathological data for patients with evidence of ULI who were intentionally diagnosed with AIH and underwent initial liver biopsy. Additional exclusion criteria were age < 18 years, acute liver failure, and clinical evidence of viral hepatitis. Further, people with certain types of cancer, heart failure, genetic metabolism, and other factors that lead to liver damage were also excluded. Some cases received drug treatment and follow-up, and they were classified as AIH and non-AIH according to the diagnostic criteria for AIH developed by the IAIHG in 1999. In our study, AIH was defined based on a pre-treatment total score > 15 or a post-treatment total score > 17.

### Data collection

For the collection of noninvasive clinical data and liver biopsy data, we mainly refer to the diagnostic criteria for AIH developed by the IAIHG in 1999 and supplement some projects by combining our clinical diagnosis, treatment experience, and literature review. These noninvasive clinical characteristics include age, gender, liver function (total bilirubin, alanine aminotransferase, aspartate aminotransferase, g-glutamyl transferase, alkaline phosphatase, fibrinogen (FBG)), AIH-related autoantibodies (AA) (anti-nuclear antibody, anti-smooth muscle antibody, anti-liver-kidney microsomal antibody, and anti-soluble liver antigen antibody), percentage of gamma globulin (GGP), white blood cell, red blood cell, and platelet. Multiple researchers conducted data collection and analysis.

### Study design and statistical analysis

All participants stratified by the training and validation cohort were presented as the means (standard deviations) or medians (interquartile ranges) for continuous variables and as frequencies (percentages) for categorical variables. First, we used univariate and multivariate logistic regression analysis to acquire the risk factors and their determination coefficients for AIH. In the model-development phase, we performed the best selection process and formulated a nomogram in the training cohort according to the Akaike information criterion. Second, the nomogram was externally assessed in the validation cohort. We operated the receiver operating characteristic curve, calibration plot, and decision curve analysis curve to report the discrimination, accuracy, and clinical utility of the novel nomogram model. Third, we calculated the optimal cutoff value of diagnosis using Youden’s index and directly presented the sensitivity, specificity, and accuracy to evaluate the nomogram model in the validation cohort, compared with the 2008 scoring criteria. IBM© SPSS 26, Stata 15 (64-bit), and R × 64 4.2.1 were used for all analyses; *p* < 0.05 was considered significant, and all statistical tests were two-tailed.

## Results

### Study population and baseline characteristics

A total of 76 individuals were excluded because they fulfilled our exclusion criteria. Overall, 127 and 125 patients were included in the final analysis as the training and validation cohorts, respectively. Table 1 shows the comparison of characteristics between training and validation cohorts. Except for FBG, GGP, and percentage of AIH, other variables are not statistically different between the two groups. These differences in FBG and GGP between the two groups may be due to the smaller number of AIH in the validation group. Table 2 shows the comparison of AIH and non-AIH in the training cohort. In the univariate logistic regression, compared with non-AIH patients, AIH patients were older, had higher levels of alanine aminotransferase, aspartate aminotransferase, and GGP, and had lower levels of FBG. What is more, AA are often positive in AIH patients.

**Table 1:** Comparison of factor characteristics between training set and validation set (n = 252).

Variables	Training set (n = 127)	Validation set (n = 125)	*p*-value
Age (years)	52 (41–59)	55 (42–62)	0.115
Gender			
Male (%)	33 (25.98%)	36 (28.80%)	0.616
Female (%)	94 (74.02%)	89 (71.20%)	
TBIL (mmol/L)	22.5 (13.2–56.8)	23.1 (13.9–47.55)	0.850
ALT (U/L)	210 (75–511)	151 (61–420)	0.157
AST (U/L)	135 (68–431)	123 (65–313)	0.746
GGT (U/L)	163 (66–320)	162 (80–268)	0.789
GGP (%)	20.5 (16.9–24.1)	17.7 (14.1–20.8)	< 0.001**
ALP (mmol/L)	134 (77–190)	150 (99–212)	0.052
ALP/ALT	0.31 (0.13–1.21)	0.41 (0.15–1.06)	0.738
FBG (g/L)	2.22 (1.85–2.77)	2.56 (2.16–2.96)	< 0.001**
WBC (10^9/L)	4.78 ± 1.71	4.88 ± 1.48	0.628
RBC (10^12/L)	4.26 ± 0.57	4.25 ± 0.57	0.916
PLT (10^9/L)	173 ± 69	174 ± 71	0.918
AA			
Positive (%)	71 (55.90%)	58 (46.40%)	0.131
Negative (%)	56 (44.10%)	67 (53.60%)	
Diagnosis			
AIH (%)	68 (53.54%)	48 (38.40%)	0.016*
Non-AIH (%)	59 (44.46%)	77 (61.60%)	

Data are shown as the means ± SD, median (interquartile range) or frequencies (%).

*TBIL* total bilirubin, *ALT* alanine aminotransferase, *AST* aspartate aminotransferase, *GGT* g-glutamyl transferase,*GGP* percentage of gamma globulin,*ALP* alkaline phosphatase, *FBG* fibrinogen, *WBC* white blood cell, *RBC* red blood cell, *PLT* platelet, *AA* AIH-related autoantibodies, *AIH* autoimmune hepatitis.

*p < 0.05, **p < 0.01.

**Table 2:** Comparison of AIH and non-AIH in the training set (n = 127).

Variables	AIH patients (n = 68)	Non–AIH patients (n = 59)	*p*-value
Age (years)	55 (45–60)	50 (38–55)	0.002*
Gender			
Male (%)	13 (19.12%)	20 (33.90%)	0.058
Female (%)	55 (80.88%)	39 (66.10%)	
TBIL (mmol/L)	24.2 (12.3–55.4)	22.3 (13.2–58.7)	0.915
ALT (U/L)	247 (109–517)	155 (41–404)	0.037*
AST (U/L)	224 (88–458)	97 (33–246)	0.002*
GGT (U/L)	177 (74–355)	154 (45–278)	0.133
GGP (%)	22.15 (18.7—28.2)	17.2 (15.8–20.9)	< 0.001**
ALP (mmol/L)	129 (83–170)	108 (70–199)	0.503
ALP/ALT	0.24 (0.13–0.79)	0.60 (0.11–2.00)	0.074
FBG (g/L)	2.20 ± 0.58	2.43 ± 0.58	< 0.001**
WBC (10^9/L)	4.75 (3.70–5.78)	4.50 (3.60–5.50)	0.503
RBC (10^12/L)	4.24 ± 0.53	4.27 ± 0.63	0.757
PLT (10^9/L)	173 ± 67	174 ± 72	0.957
AA			
Positive (%)	52 (76.47%)	19 (32.20%)	< 0.001**
Negative (%)	16 (23.53%)	40 (67.80%)	

Data are shown as the means ± SD, median (interquartile range) or frequencies (%).

*TBIL* total bilirubin, *ALT* alanine aminotransferase, *AST* aspartate aminotransferase, *GGT* g-glutamyl transferase,*GGP* percentage of gamma globulin,*ALP* alkaline phosphatase, *FBG* fibrinogen, *WBC* white blood cell, *RBC* red blood cell, *PLT* platelet, *AA* AIH-related autoantibodies, *AIH* autoimmune hepatitis.

*p < 0.05, **p < 0.01.

### Development of an AIH-predicting nomogram

**Figure 1:**
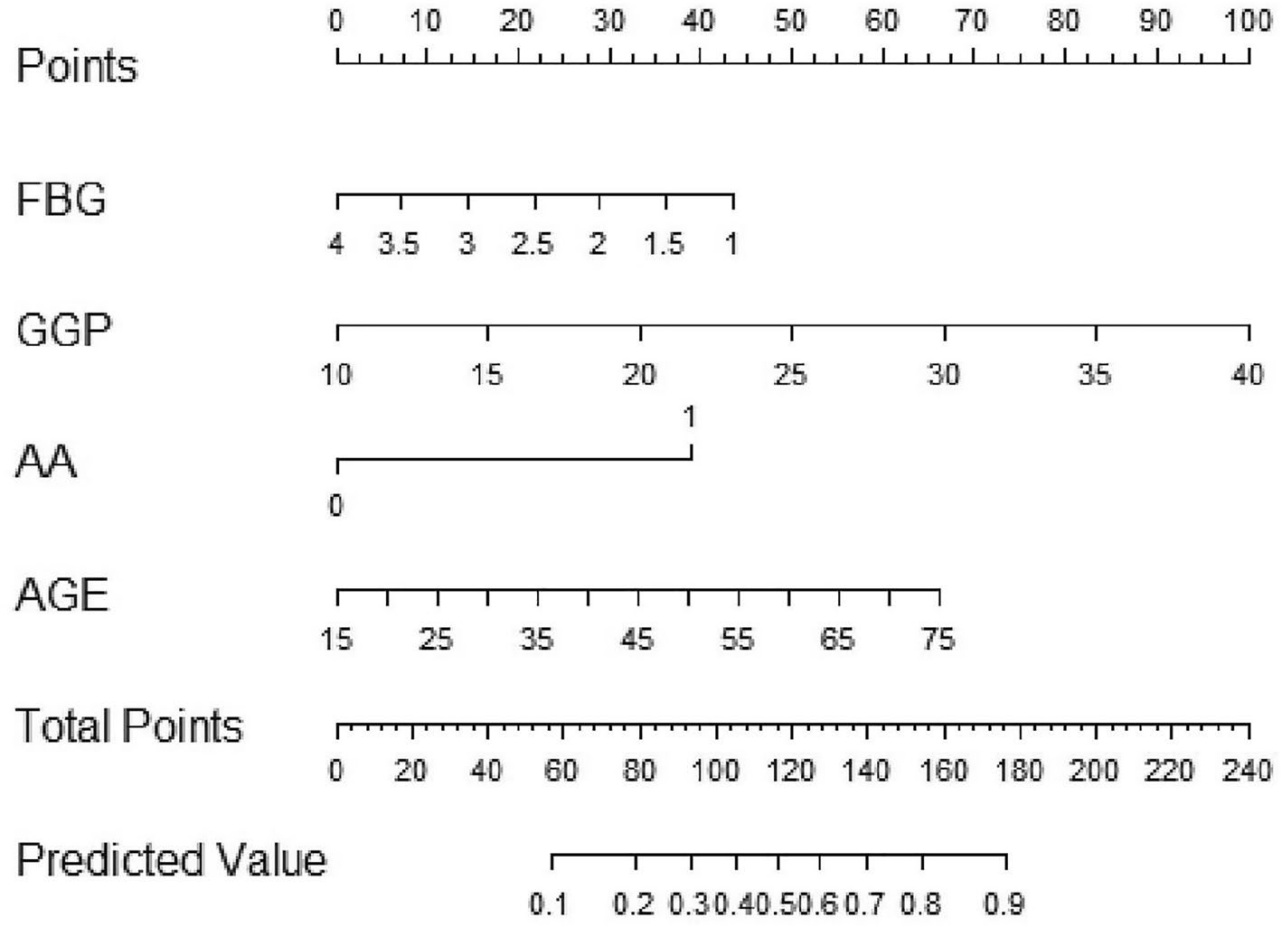
Nomogram for predicting the risk of AIH based on age, FBG, GGP, and AA. An upward vertical line was drawn from each variable axis to get the points of each variable. Calculating the sum of each variable point, and drawing a downward vertical line from the total points axis to obtain the risk of AIH. Example of use: A 50-year-old female is positive for AA with a FBG level of 3.2 g/L and a GGP of 22.5%. The corresponding points of age, FBG, GGP and AA are 40, 13, 42, and 38 respectively, with total points of 133, and the risk of AIH was about 0.64. *GGP* Percentage of gamma globulin, *FBG* fibrinogen, *AA* AIH-related autoantibodies, *AIH* autoimmune hepatitis.

Based on the Akaike information criterion, we performed a nomogram of the stepwise model including age, FBG, GGP, and AA to predict the risk of AIH by using multivariate logistic regression (Fig. 1). To estimate an individual’s risk of AIH, the value is located on each variable axis. A vertical line is drawn from that value to the top point scale to determine how many points are assigned to that variable value. Then the points from each variable value are added. The sum is located at the total points scale and is vertically projected onto the bottom axis, thus obtaining a personalized risk of AIH.

### Validation of the nomogram model for AIH

**Figure 2:**
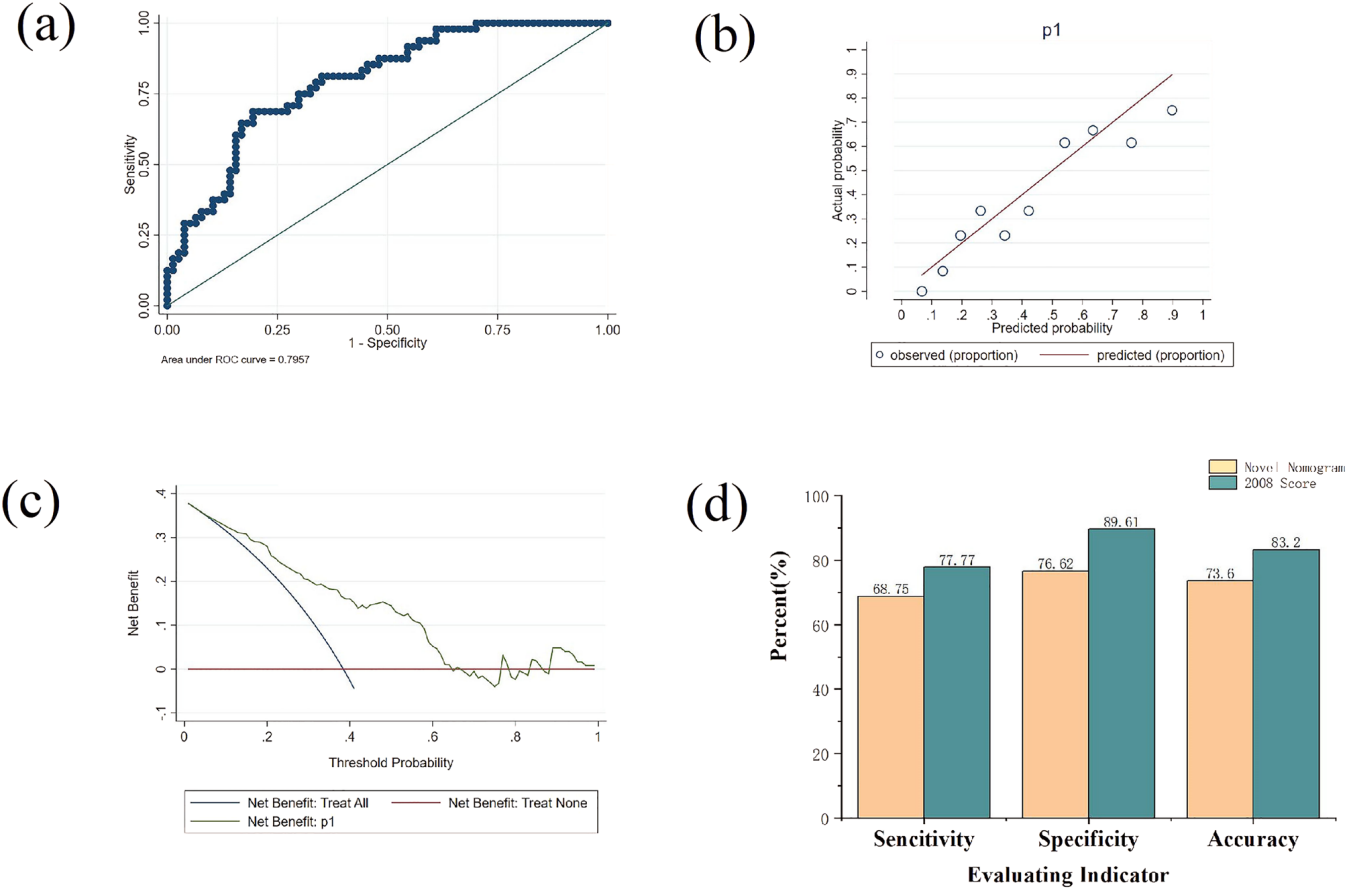
Validation of the nomogram model for AIH in the validation cohort. (**a**) The areas under the curve for the validation cohorts were 0.796. (**b**) The calibration plot suggested that the model had an acceptable accuracy (*p* > 0.05). (**c**) The decision curve analysis suggested that the model had great clinical utility if the value of probability was 0.45. (**d**) The sensitivity, specificity, and accuracy of our model compared with the 2008 International Autoimmune Hepatitis Group simplified scoring system.

We used Youden’s index to determine the cutoff value in the training cohort, and the calculated cutoff value was 0.45. The areas under the curve were 0.796 in the validation cohort (Fig. 2a). As the areas under the curve of the validation cohort were close to 0.8, the discrimination of our model was not bad. To assess the predicted probability of the model, the patients were divided into ten groups, and the calibration map of the training cohort was constructed. The calibration plot (Fig. 2b) revealed the accuracy of our model, the fitted line coincided with the 45-degree line, which means the prediction was roughly accurate (*p* > 0.05). In addition, we established decision curve analysis in validation cohorts (Fig. 2c) to assess the clinical utility. We could properly draw the conclusion that this model showed good clinical utility if the value of probability was 0.45.

Also, we measured directly the accuracy of our model compared with the IAIHG simplified scoring system in 2008. In the validation cohort, based on the cutoff value, we succeeded in predicting 33 people out of the 48 real AIH patients and 59 people out of the 77 real non-AIH patients, which with a sensitivity of 68.75%, specificity of 76.62% and accuracy of 73.60%. While we diagnosed the validated population by using the 2008 diagnostic criteria, 35 of 48 AIH patients and 69 of 77 non-AIH patients were successfully predicted, the sensitivity of prediction results was 77.77%, the specificity was 89.61% and the accuracy was 83.20%. The outcome is shown in Fig. 2d. Although our model did not include a pathology factor, the predictive power was not much different from the 2008 IAIHG simplified scoring system.

## Discussion

We developed a nomogram to predict the risk of AIH using four risk factors (Age, FBG, GGP, and AA). The external validation results showed predictive value making it as a useful tool in the clinic. We have designed a promising model that can predict the risk of AIH with noninvasive data. The intuitiveness of visualization, which is the merit of the nomogram, is able to understand the risk factors for AIH in ULI patients.

Liver biopsy is one of the main parameters in all three versions of the diagnostic criteria for AIH developed by the IAIHG^10–13^. The simplified diagnostic criteria in 2008 has been widely used^3–7^. However, some ULI patients probably refuse to undergo the liver biopsy examination due to the fear of invasiveness, especially in early stages. It would undoubtedly further reduce the practical application value of the 2008 scoring criteria of AIH including liver biopsy. Based on this phenomenon, we established a model to predict the risk of AIH with noninvasive data. Our model provided an extra option for these patients with scary of liver biopsy after ruling out factors such as hepatitis, alcohol, drugs, and patients with recurrent chronic liver damage. The validated population in our model showed good discrimination, accuracy, and clinical utility by estimating the risk of AIH. Moreover, we compared the sensitivity, specificity, and accuracy of our model and the 2008 simple diagnostic standard in predicting AIH. The results show that our model has a little lower in these three indexes (68.75% vs. 77.77%; 76.6% vs. 89.61%; 73.6% vs. 83.2%). Meanwhile, this model provides a valuable supplement to the IAIHG simplified scoring system in 2008. If our model predicts a high risk of AIH, we can also persuade patients, especially those who are in the hesitant stage, to do a liver biopsy.

In our study, we found that a new indicator, FBG, is associated with AIH. Clinically, we also observed that FBG in most AIH patients is lower than that of normal people. A study showed that the process of fibrinogen-degradation products had a negative effect on AIH, which is roughly consistent with our paper’s conclusion^16^. Recent studies^17^ discovered that FBG promotes cell aggregation and fibrin accumulation, improving liver function in organoids. And he also found that FBG improves liver function by activating the wnt/β-certain signaling pathway in the microenvironment. It is easy to understand that FBG represents the synthetic function of the liver. When patients with AIH have impaired synthetic function of the liver, FBG will decrease. Of course, there is still much need to verify the relationship between FBG and AIH.

AIH can occur at any age according to its mechanism, and we found that age is a predictive risk factor for AIH. According to some studies^18,19^, the distribution of age at the onset of AIH was thought to be bimodal, with peaks around puberty and between the fourth and sixth decades of life. It should be noted that the age of the AIH population in this study was also approximately 40–60 years. Therefore, the recommendation is to consider patients of all ages who may have AIH, but with an emphasis on patients between the ages of 40 and 60. As noted in the “Brighton report”, several other autoantibodies are of relevance to AIH, namely, those reacting with the hepatic asialoglycoprotein receptor^20^, but those are still only available in a few specialized laboratories. For autoantibodies, we chose anti-nuclear antibody, anti-smooth muscle antibody, anti-liver-kidney microsomal antibody, and anti-soluble liver antigen antibody as our analysis according to the 1999 criteria of AIH. And we did not stratify the antibody results into different levels because we considered that the current detection methods and criteria in many hospitals are not completely unified. However, the higher the antibody titer, the more likely it is to diagnose AIH. Moreover, in the study, it was found that more female ULI patients were diagnosed with AIH than men. However, there was no statistically significant difference between the two genders, possibly due to small sample size.

The specific mechanisms of autoimmune diseases in women are not completely understood. Recent studies^21,22^ have shown that the role of sex hormones such as estrogen, testosterone, and progesterone in the immune response. Estrogen receptors can regulate many aspects of T cell function, including T cell activation, proliferation, and survival. Regulatory T cells are a type of T cell family that can help effectively suppress the occurrence of autoimmune diseases. When women reach menopause and reproductive age, estrogen levels change dramatically, which can damage the immune system and lead to autoimmune diseases. This might also explain why AIH can occur at any age but usually peaks around puberty and between the ages of 40 and 60. Therefore, we should pay special attention to female patients with ULI during clinical work. In addition to considering AIH, we also need to consider drug-induced liver injury, primary biliary cirrhosis, primary sclerosing cholangitis, alcoholic liver disease, and nonalcoholic steatohepatitis, etc. in patients with ULI, because they may have similar clinical and pathological manifestations.

There are some problems in the research that we must mention. Firstly, the inherent limitations of the cross-sectional case–control study design failed to establish a causal relationship. In future research, we will continue to train and validate our model by conducting subsequent larger multicenter studies and external validation studies. Secondly, the identification of the drug-induced liver injury and AIH is still difficult, and liver histology examination plays an important role in detection. Our model without liver biopsy is hard to identify drug-induced liver injury. Therefore, for patients taking minocycline, nitrofurantoin, infliximab, and other drugs that are obviously harmful to the liver, if there are no obvious contraindications, a liver biopsy is recommended for further identification. Finally, the case data we collected covered a span of 15 years and involved three different hospitals, 252 patients were included in this study and the sample size was not relatively large. This is due to the low prevalence of AIH and the fact that some patients do not want to do liver biopsy due to its invasiveness, resulting in a reduced number of subjects to be tested. That is why we aim to operate another supplementary diagnostic method of AIH without a liver biopsy. However, larger samples are required in future studies to further evaluate the functional validation of this model.

In conclusion, the new model without liver biopsy we developed shows great risk predictive value for ULI patients who are likely to be diagnosed with AIH. Furthermore, this model is a valuable supplement to the 2008 IAIHG simplified scoring system. The model proposed in this paper could be valuable and clinically meaningful.

## Data Availability

All datasets generated for this study are included in the article.
